# COVID-19 With an Initial Presentation of Intraperitoneal Hemorrhage Secondary to Spontaneous Splenic Rupture

**DOI:** 10.7759/cureus.15310

**Published:** 2021-05-28

**Authors:** Mohammed Knefati, Ismail Ganim, Jozef Schmidt, Abhilash Makkar, Stephanie Igtiben, Eric Landa, Ahmad Tarawneh, Courtney Hicks, Stacy Zimmerman, Suporn Sukpraprut-Braaten

**Affiliations:** 1 Internal Medicine, Unity Health, Searcy, USA; 2 Pulmonary and Critical Care Medicine, Unity Health, Searcy, USA; 3 Infectious Diseases, Unity Health, Searcy, USA; 4 Research, Unity Health, Searcy, USA

**Keywords:** covid-19, atraumatic splenic rupture, intra-abdominal collection, gastro-intestinal, hemato

## Abstract

The WHO declared coronavirus disease 2019 (COVID-19) a global pandemic in early 2020. As the pandemic has continued to evolve over a period of several months, many cases of unusual presentations are now emerging, which pose a greater challenge for physicians in terms of quickly identifying COVID-19 patients based on initial signs and symptoms. In this report, we present one such unusual presentation in a patient with sudden intraperitoneal hemorrhage and spontaneous splenic rupture with COVID-19 as the likely etiology and contributing factor.

The patient was a 75-year-old Caucasian woman who presented to the emergency department (ED) with complaints of severe left-sided abdominal pain for several days without any preceding trauma. A CT of the abdomen/pelvis revealed a large amount of fluid in the abdomen, which raised suspicion of bleeding. An exploratory laparotomy revealed splenic rupture with hemoperitoneum, and the patient subsequently underwent an emergent splenectomy. The patient’s COVID-19 antigen test returned positive during the surgery and was subsequently confirmed with a polymerase chain reaction (PCR) test.

COVID-19 has been found to result primarily in respiratory symptoms through its ability to invade endothelial cells via angiotensin-converting enzyme 2 affinity. It is speculated that this mechanism may cause a predisposition to micro-thromboses, which can eventually lead to manifestations such as large lymphoid organ thrombosis. Based on this case presentation and the evolving literature on severe acute respiratory syndrome coronavirus 2 (SARS-CoV-2), spontaneous splenic rupture is an emergent differential diagnosis that should be considered in COVID-19 patients presenting with gastrointestinal complaints such as abdominal pain and nausea.

## Introduction

Severe acute respiratory syndrome coronavirus 2 (SARS-CoV-2) is the cause of the ongoing coronavirus disease 2019 (COVID-19) global pandemic. As of October 31, 2020, there have been more than 45.7 million cases of COVID-19 globally with more than 1.19 million deaths [1]. Although commonly manifested by fever (up to 90% of hospitalized patients) and respiratory symptoms (53-80% of hospitalized patients) [2], the current estimates of asymptomatic infection range between 4-41% [3].

Many cases of unusual presentations of COVID-19 have also been reported, which poses a greater challenge for physicians as they may find it difficult to promptly identify patients of COVID-19 based on initial clinical signs and symptoms. One such presentation involves abdominal pain secondary to hemoperitoneum. Although splenic rupture is an emergency condition often secondary to trauma, here we present a patient with sudden intraperitoneal hemorrhage and spontaneous splenic rupture with COVID-19 as the likely etiology and contributing factor [4].

Splenic rupture is an acute emergency typically occurring due to trauma but can also occur in an atraumatic setting in rare cases. Atraumatic splenic rupture, which is routinely reported as spontaneous, has an approximate incidence rate of 0.1-0.5% [5,6]. Typically, atraumatic etiologies include neoplastic diseases (30%) and infectious (27%), inflammatory (20%), treatment-related (9%), mechanical (7%), and idiopathic causes [7].

Most notable among the infectious etiologies of spontaneous splenic rupture are infectious mononucleosis (40%) and malaria (17%) [7,8]; however, in recent years, reports of respiratory illness-associated splenic rupture have appeared in the literature. While cough-associated spontaneous splenic rupture has been discussed in the literature [7,9-11], pathologic splenic changes were noted during the 2003 SARS outbreak, which showed extensive damage to normal splenic architecture and altered immune cell numbers [12]. Analysis of spleens and immune cell distribution in patients with SARS-CoV-2 continues to reveal significant abnormalities in lymphocyte, granulocyte, and monocyte distributions [13]. Of note, exogenous administration of granulocyte colony-stimulating factor (G-CSF) has been associated with spontaneous splenic rupture [14]. Interestingly, G-CSF has been implicated in the pathogenesis of SARS-CoV-2 and identified as a driving factor of immune system dysregulation [15,16]. This marks one of the major differences between SARS-CoV-2-related splenic ruptures and other infectious and hematologic etiologies - these result in splenomegaly in approximately 55% of cases [17]. Several case reports on spontaneous splenic rupture in patients with SARS-CoV-2 [17,18] as well as splenic thromboembolic disease have surfaced within the last year [4,19-21]. We present a case of an elderly woman whose initial COVID-19 presentation at the hospital involved atraumatic/spontaneous splenic rupture in the absence of respiratory symptoms.

## Case presentation

A 75-year-old Caucasian woman with a past medical history of hypertension, hyperlipidemia, and atrial fibrillation with chronic anticoagulation on apixaban was brought to the emergency department (ED) due to severe abdominal pain. The patient had initially presented to her primary care physician (PCP) two days prior with nausea and emesis. The following day, she had developed left-sided abdominal and shoulder pain. She described the pain as constant, sharp, stabbing, and radiating diffusely across the abdomen. The pain, nausea, and vomiting had progressively worsened over the weekend, and hence she had decided to go to the hospital. She denied any fever, chills, cough, dyspnea, chest pain, or palpitations. She also denied any recent trauma. The patient did state that her husband had been having low-grade fevers at home.

The patient's vitals on physical examination were as follows: temperature (T) of 98.1 ^o^F, blood pressure (BP) of 83/54 mmHg, a pulse rate of 86 beats per minute, respiratory rate (RR) of 18 breaths per minute, O_2_ saturation of 94% on room air, and pain rated as 10/10 in severity. She was in moderate pain and distress. Her heart sounds were regular and rhythmic, and peripheral perfusion was normal. Lungs were clear to auscultation without wheezing, rhonchi, or crackles. The abdomen was tender to palpation in the left upper and lower quadrants. Bowel sounds were hypoactive. There were no pulsatile masses, and the skin was without any rashes.

Her laboratory workup was significant for a positive COVID-19 antigen test, and her polymerase chain reaction (PCR) test was also positive. Her ferritin level was 317.9 mcg/L; C-reactive protein (CRP) was 0.4 mg/dl at presentation but increased to 13.0 mg/dl within two days. Other lab findings were as follows: D-dimer of 0.81 mcg/mL FEU, WBC of 9.0 x 1000/mm^3^, hemoglobin (HGB) of 12.3 g/dL, hematocrit (HCT) of 37.0%, platelets (PLT) of 204 x 1000/mm^3^, sodium (Na) of 138 MM/L, potassium (K) of 3.6 MM/L, chloride (Cl) level of 104 MM/L, CO₂ of 21 MM/L, glucose (GLU) of 163 mg/dl, blood urea nitrogen (BUN) of 23 mg/dl, creatinine (Cr) of 1.0 mg/dl, haptoglobin of 179 mg/dl, and procalcitonin of 0.32 ng/mL.

**Figure 1:**
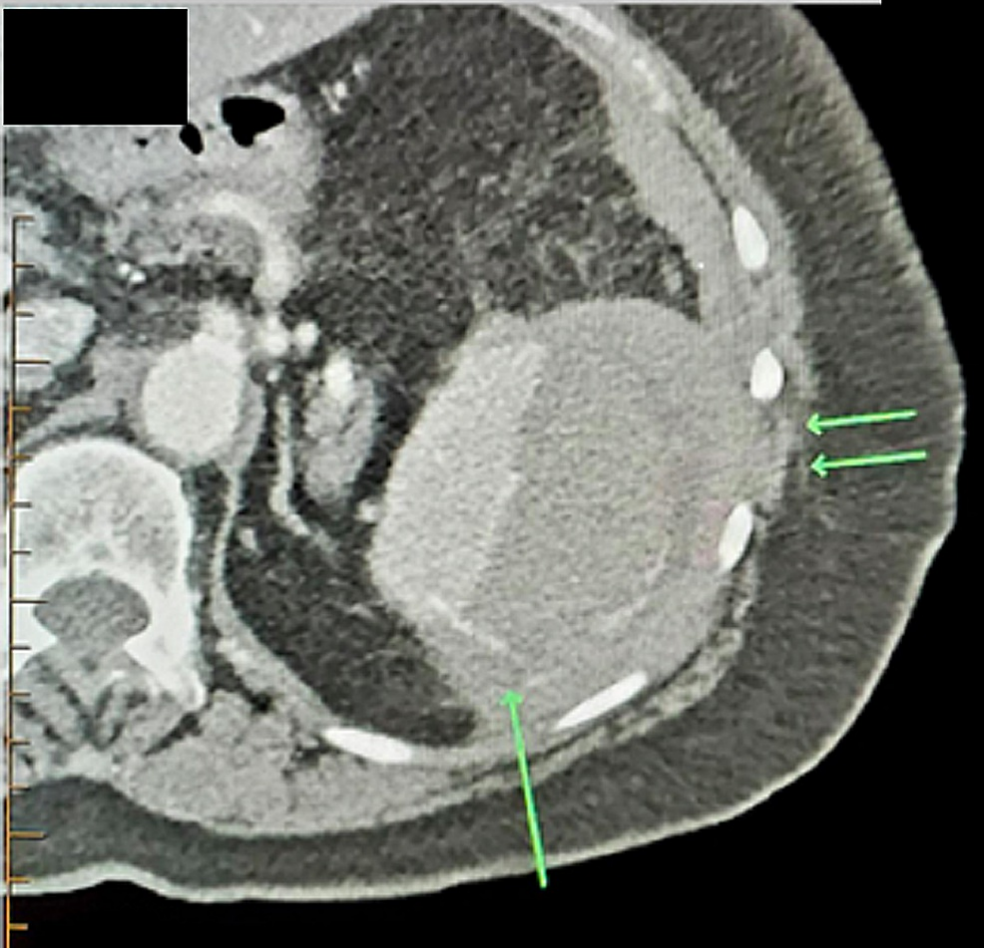
CT abdomen/pelvis of the patient’s spleen The image shows active extravasation (single arrow) and hematoma (double arrow) CT: computed tomography

A CT of the abdomen/pelvis obtained in the ED revealed a large subcapsular fluid collection measuring 3.2 cm in thickness, surrounding a small spleen and indicating a subcapsular hematoma (Figure 1). The spleen was noted to be small in size, measuring 7.8 cm longitudinally. The patient received IV Kcentra 3500 mg, IV Flagyl 500 mg, 2L lactated Ringer's (LR) bolus, IV morphine 4 mg, and IV Zofran 4 mg while in the ED.

**Figure 2:**
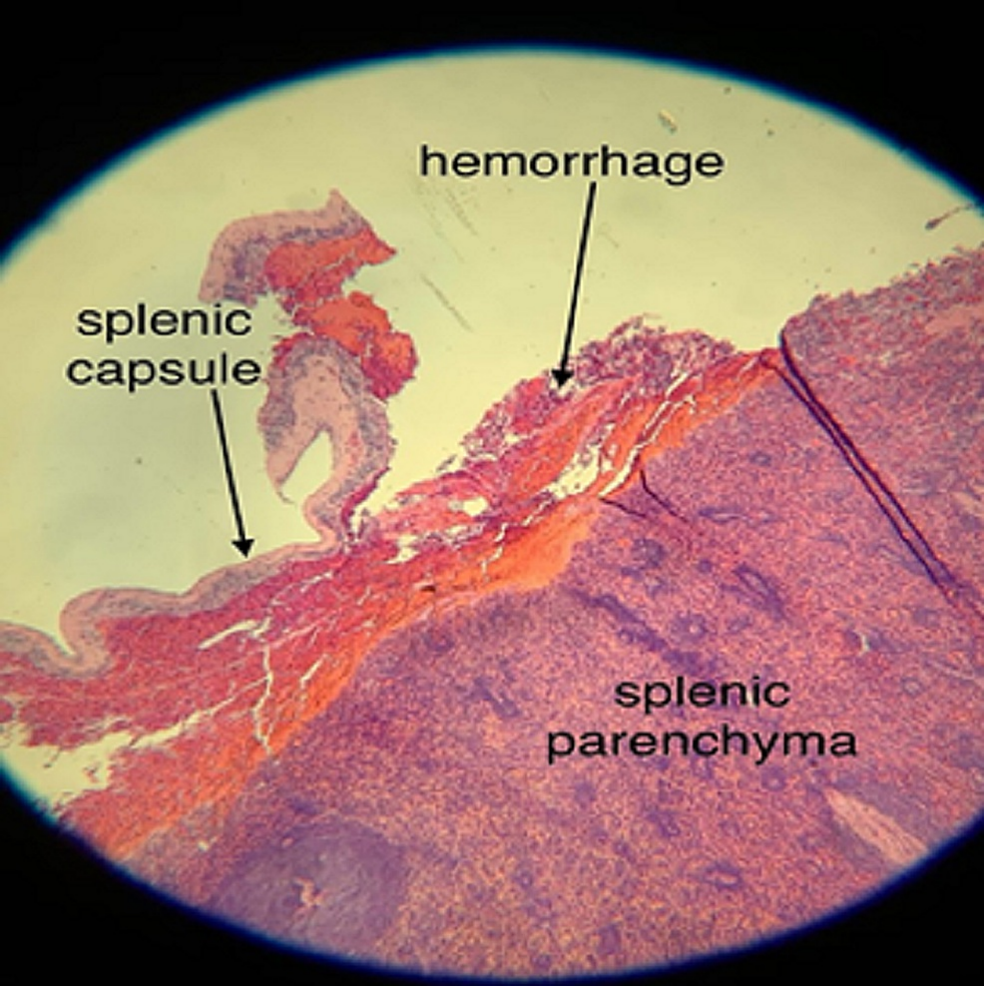
Microscopic image from Pathology lab revealing capsular rupture with an associated area of subcapsular hemorrhage

General Surgery was consulted, and the patient was taken to the operating room (OR) emergently for exploratory laparotomy. In the OR, a ruptured spleen with hemoperitoneum was discovered and a splenectomy was successfully performed without complications. The patient’s COVID-19 antigen test returned positive during the surgery and was subsequently confirmed with PCR. The patient’s spleen was sent to the Pathology lab and, under microscopic analysis, an area of capsular rupture measuring 3.5 x 1 cm was noted, with an associated area of subcapsular hemorrhage (Figure 2).

The patient's background information was unremarkable. She had not experienced any recent trauma; there was no significant family history, or any alcohol, tobacco, or illicit drug use. The only known risk factor the patient presented with was a positive COVID-19 test. The patient tolerated the surgery well and recovered without serious complications under ICU care.

## Discussion

COVID-19 is associated with a wide range of symptoms including fever (70-90% of hospitalized patients), dry cough (60-86% of hospitalized patients), flu-like symptoms [22-29], and new loss of taste or smell (64-80% of all patients) [30-32]. Interestingly, there have also been many reports associating COVID-19 with thrombotic diseases such as deep vein thrombosis, pulmonary embolism, stroke, and disseminated intravascular coagulation (DIC).

Although the mechanism is not fully understood, the condition is thought to cause respiratory symptoms by its ability to invade pulmonary endothelial cells through angiotensin-converting enzyme 2 affinity [22-25]. It is speculated that this endothelial dysfunction may cause a cascade of the activation of the inflammatory response, including components such as complement activation, leukocyte demargination, as well as activation of the immune response, which seems to lead to a predisposition to micro-thrombosis [26]. The degree of micro-thrombosis may impact the mortality rates of COVID-19 and is usually manifested subclinically without any acute intra-abdominal hemorrhagic indication [30,31].

In documented cases presenting with acute intra-abdominal manifestations such as spontaneous hemoperitoneum, patients are typically found to manifest respiratory symptoms or reveal labs significant for lymphopenia and elevated d-dimer levels, which represent inappropriate activation of coagulation leading to visceral infarction [32].

Interestingly, this COVID-19 patient presented without any respiratory symptoms and her coagulation parameters (negative D-dimer and normal platelet count) deviated from those seen in prior studies. Furthermore, on pathological examination, the patient’s spleen measured 7.8 cm in length, which is smaller than the average size (11.2 cm) for women of this age range, and much smaller than what would be expected in viral infections typically associated with spontaneous splenic rupture, such as infectious mononucleosis and malaria, which often present with splenomegaly secondary to lymphocytic infiltration [33-37].

The precise etiology and mechanism for this patient’s splenic rupture are still unclear. However, as similar cases continue to be reported in the literature, it is clear that presentations of abdominal pain in the setting of COVID-19 warrant further evaluation and that COVID-19 should be considered as another possible infectious cause of the spontaneous splenic rupture. Finally, there is currently much speculation regarding the physiological mechanism(s) by which COVID-19 may affect inflammatory and immune responses leading to coagulopathy and thromboses, and it is important to further investigate these issues as well given their clinical implications.

## Conclusions

COVID-19 appears to physiologically manifest in a variety of multi-systemic phenomena, and clinicians must maintain a high degree of suspicion about the possibility of COVID-19 when evaluating patients with unusual symptoms, especially in relation to the possibility of micro-thromboses or primary lymphoid involvement. Based on this case presentation, spontaneous splenic rupture is an emergent differential diagnosis that should be considered in COVID-19 patients presenting with gastrointestinal complaints such as abdominal pain and nausea.
